# Clinical efficacy and safety evaluation of dexmedetomidine hydrochloride nasal spray for sedation before examinations in children aged 2 to 6 years: protocol for a randomized controlled trial

**DOI:** 10.3389/fped.2026.1942624

**Published:** 2026-09-24

**Authors:** Xiaoling Wang, Xiong Li, Xiaoping Lei, Wenbin Dong

**Affiliations:** 1 Jinan University, Guangzhou, China; 2 Department of Neonatology, Children’s Medical Center, the Affiliated Hospital of Southwest Medical University, Luzhou, China; 3 Department of Urology, The Affiliated Hospital of Southwest Medical University, Luzhou, China

**Keywords:** children, chloral hydrate, dexmedetomidine, nasal spray, pediatric sedation, protocol, randomized controlled trial

## Abstract

**Background:**

Children aged 2 to 6 years are prone to fear and resistance during medical examinations, necessitating sedation assistance. Traditional sedation modalities have limitations such as inconvenient operation, slow onset, or respiratory depression. Dexmedetomidine hydrochloride, a highly selective *α*₂-adrenergic receptor agonist, exerts sedative, analgesic, and anxiolytic effects with minimal respiratory and circulatory impacts. Its nasal spray is rapidly absorbed via the mucosa, avoiding the first-pass effect, and is thus suitable for young children. This study compares intranasal dexmedetomidine hydrochloride with oral chloral hydrate to evaluate the former's clinical efficacy and safety for pre-examination sedation in young children.

**Methods:**

This is a single-center, prospective, randomized controlled trial conducted at the Children's Medical Center of the Affiliated Hospital of Southwest Medical University. A total of 248 children aged 2–6 years with ASA physical status I/II requiring sedation for outpatient examinations will be enrolled and randomized 1:1 to receive dexmedetomidine hydrochloride nasal spray (2*μ*g/kg, experimental group) or oral chloral hydrate syrup (50 mg/kg, control group) via computer-generated random sequences. Vital signs will be monitored continuously; sedation onset/duration, MOAA/S (sedation depth), and Steward (recovery status) scores will be recorded. Adverse events will be observed to compare sedation success rates and safety between groups.

**Ethics and dissemination:**

This study has been approved by the Medical Ethics Committee of the Affiliated Hospital of Southwest Medical University (Approval No. KY2025368). Results will be disseminated via peer-reviewed publications and conference presentations.

**Clinical Trial Registration:**

https://www.chictr.org.cn/showproj.html?proj= 320640, ChiCTR2600126783.

## 1 Introduction

Children aged 2 to 6 years have immature neuropsychological development and are prone to fear and uncooperative behavior when undergoing outpatient diagnostic examinations such as magnetic resonance imaging (MRI) and computed tomography (CT). Sedation assistance is often required to ensure the smooth progress of examinations (1, 2). Currently commonly used sedative drugs such as chloral hydrate, ketamine and midazolam may cause gastrointestinal irritation, respiratory depression or potentially affect neurological development (3–6). Traditional sedation methods have disadvantages including delayed onset, low bioavailability and difficult regulation of sedation depth (7–9).

Transmucosal drug delivery achieves rapid drug absorption through the submucosal vascular network, which can bypass the hepatic first-pass metabolism and directly act on the central nervous system through the “nose-brain pathway”, becoming the preferred route of non-invasive drug delivery (10–12). Dexmedetomidine hydrochloride is a highly selective *α*₂-adrenergic receptor agonist with both sedative and analgesic effects, and has minimal impact on the respiratory and circulatory systems with a bioavailability of 65%–80%, making it a commonly used preoperative sedative in pediatrics (13). Large-sample studies have shown that its adverse event rate is low, and reactions such as bradycardia are mostly within the safe range without the need for drug intervention (14, 15). In addition, intranasal administration can avoid the discomfort associated with injection and is easy to operate, with sedation induced within 30 min. It has no hepatic first-pass effect or gastrointestinal irritation, and has a higher sedation success rate and faster onset of action than oral chloral hydrate in pediatric CT/MRI examinations, with good safety in children of all ages (including newborns) (16–22).

In view of the problems of uneven drug distribution, easy crying and coughing caused by the existing intranasal instillation administration, nasal spray can achieve quantitative and accurate drug delivery with the help of microatomization technology, improve bioavailability, reduce irritation and coughing, and the upright administration is suitable for young children (23). Therefore, this study intends to evaluate the efficacy and safety of dexmedetomidine hydrochloride nasal spray at a dose of 2μg/kg for sedation in outpatient diagnosis and treatment of children aged 2 to 6 years through a randomized controlled trial, so as to provide a reference for pediatric comfort medicine.

It should be noted that clinical practices for pediatric MRI sedation vary across countries: general anesthesia is routinely used in some settings to ensure immobility, whereas procedural sedation under anesthesiologist monitoring is the standard approach for outpatient imaging in ASA I/II children in most Chinese pediatric centers. Large-sample evidence supports the safety and efficacy of intranasal dexmedetomidine for pediatric MRI sedation (16). This study is designed to provide randomized controlled evidence for this widely practiced approach.

## 2 Methods and analysis

### 2.1 Study aim

To compare the clinical efficacy and safety of dexmedetomidine hydrochloride nasal spray and oral chloral hydrate syrup for pre-examination sedation in children aged 2 to 6 years, and to provide an optimized evidence-based sedation option for this pediatric population.

### 2.2 Study design and setting

This is a single-center, prospective, randomized controlled trial conducted at the Children's Medical Center of the Affiliated Hospital of Southwest Medical University under routine clinical conditions. The study period is from July 1, 2026 to August 31, 2027. The study is currently in the pre-recruitment stage, and no participants have been enrolled as of the submission date. The study protocol has been approved by the Medical Ethics Committee of the Affiliated Hospital of Southwest Medical University and is supervised by an independent academic committee.

### 2.3 Study participants

#### 2.3.1 Inclusion criteria

All children must meet all the following criteria to be enrolled in the trial:

1. Aged 2 to 6 years (inclusive), regardless of gender;
2. Difficulty in cooperating with examinations on their own and requiring sedation to complete relevant outpatient diagnostic procedures, including but not limited to magnetic resonance imaging (MRI), computed tomography (CT), echocardiography, auditory brainstem response (ABR), ophthalmic examinations, and pulmonary function tests;
3. ASA physical status classification I or II;
4. Legal guardians of the children have signed the informed consent form.

#### 2.3.2 Exclusion criteria

Children who meet any of the following criteria will be excluded from the trial:

1. ASA physical status classification III or above;
2. Severe rhinitis, nasal deformity and nasal mucosal damage;
3. Severe upper respiratory tract infection;
4. Cardiac arrhythmia, especially chronic arrhythmia;
5. Hepatic and renal insufficiency;
6. A history of hypersensitivity to sedative drugs;
7. General anesthesia within 2 weeks;
8. Use of sedative-hypnotic drugs (including benzodiazepines such as diazepam and barbiturates such as phenobarbital) within 1 week;
9. Mental system diseases and cognitive dysfunction;
10. Severe obesity (body weight ≥ 50% higher than the standard body weight);
11. Loss to follow-up;
12. Refusal to participate in this study;
13. Other circumstances deemed unsuitable for participation in this clinical trial by the researchers.

### 2.4 Sample size calculation

A total of 248 children aged 2 to 6 years who require sedation assistance to complete outpatient diagnostic and therapeutic procedures will be enrolled in this study to evaluate the efficacy and safety of dexmedetomidine hydrochloride. The sample size estimation is based on the following:

1. For the efficacy endpoint: with a one-sided *α* of 0.05 and a one-sided power of 80%, the sedation success rate of the chloral hydrate group is 65% as a reference, and the expected sedation success rate of the dexmedetomidine hydrochloride nasal spray group is 85% (24). A 1:1 superiority test was performed using PASS software, and a total of 214 cases were calculated. Considering an approximate 10% dropout rate, a final total of 238 cases are needed (119 cases in each group).
2. For the safety endpoint: according to literature reports (25), the maximum incidence of adverse events of oral chloral hydrate is about 10.3%, and that of dexmedetomidine hydrochloride is about 13%. With a two-sided *α* of 0.05 and a two-sided power of 80%, and an equivalence margin of 15%, a 1:1 equivalence test was performed using PASS software, and a sample size of 222 cases was calculated. Considering a 10% dropout rate, a final total of 248 cases are needed (124 cases in each group).

The 15% equivalence margin was predefined as the absolute risk difference in adverse event incidence between groups. It was chosen because sedation-related adverse events in paediatric non-invasive imaging are mostly transient and mild-to-moderate, resolving without intervention (25), and because our paediatric anaesthesiology team considered 15% the largest clinically acceptable difference; this margin is consistent with the 10%–15% range commonly adopted in paediatric procedural sedation trials.

Comprehensively considering the efficacy and safety, the final sample size of this study is set at 248 cases, based on the results of the safety equivalence test.

### 2.5 Randomization and blinding

Participants will be randomly assigned in a 1:1 ratio using a computer-generated random sequence. The random sequence will be generated by a professional statistician who is not involved in the clinical implementation of the study, and the grouping will be concealed using sealed opaque envelopes to ensure the randomness and concealment of the grouping. The random sequence and concealment process will be documented in detail and stored in a locked file accessible only to the statistician until the study is completed.

This study adopts an assessor-blinded design. Drugs are administered by designated sedation nurses who take no part in outcome assessment. Sedation depth (MOAA/S), recovery status (Steward score) and adverse events are assessed by anesthesiologists or researchers unaware of group allocation, with no access to administration records. Group allocation is coded (Group A/B) and statistical analyses are performed under blinded conditions until the analysis plan is locked. Emergency unblinding for serious adverse events follows prespecified procedures and is documented.

### 2.6 Interventions

#### 2.6.1 Pre-sedation preparation

Children will be woken up 2 h early on the examination day or their noon sleep will be cancelled. According to the 2023 American Society of Anesthesiologists (ASA) guidelines (26), routine preoperative fasting is required: fasting from starchy solid foods, liquid dairy products such as milk, and formula milk for 6 h, fasting from breast milk for 4 h, and a small amount of clear liquids (≤5 mL/kg, total volume not exceeding 300 mL) can be taken 2 h before anesthesia. All children will be administered drugs by sedation nurses 40 min before the examination in accordance with the drug instructions.

#### 2.6.2 Experimental group: dexmedetomidine hydrochloride nasal spray

Dexmedetomidine hydrochloride nasal spray used in this study is produced by Jiangsu Hengrui Pharmaceutical Co., Ltd. Before administration, nasal secretions of the children should be cleaned to keep the nasal cavity dry. The child is placed in a sitting position with the head slightly forward. The activated nasal spray pump is kept upright, the nozzle is inserted into the nostril at the same angle as the nasal cavity, and the pump body is pressed to the bottom at one time to complete unilateral or bilateral intranasal spray. The drug solution is available in two concentration specifications (Type I: 500μg/mL, 25μg per spray; Type II: 300μg/mL, 15μg per spray). A fixed weight-stratified dosing scheme is applied according to the drug label and the phase III trial regimen (27): 30μg (Type II, 15μg/spray   ×   2) for children weighing 10.7–< 19.4 kg, and 50μg (Type I, 25μg/spray  ×  2) for 19.4–≤ 28.0 kg; for other weights, Type II sprays are rounded to the nearest whole spray targeting 2μg/kg. The specification, number of sprays and actual dose will be recorded in the CRF. The entire administration operation should be completed within 2 min. This product is a single-use device, and the remaining drug solution must be disposed of in accordance with the specifications for medical waste.

#### 2.6.3 Control group: oral chloral hydrate syrup

Chloral hydrate syrup combination package is produced by Nanjing Tefeng Pharmaceutical Co., Ltd., Jiangsu Province. Take chloral hydrate concentrate, draw the clinical dose of chloral hydrate (50 mg/kg, maximum single dose 1 g) with a medicine taking syringe, inject it into the syrup (diluent), shake it vigorously more than 3 times to mix well to obtain the clinical use solution. An appropriate amount is taken orally according to the body weight, prepared and used immediately, and taken at one time. The actual administered dose (mg/kg and total mg) will be recorded in the CRF.

#### 2.6.4 Rescue sedation

Rescue sedation will be initiated if the MOAA/S score remains > 2 at 40 min after the initial administration, or if the child awakens during the examination with body movements precluding continuation. The originally assigned drug will be supplemented first: an additional 1μg/kg of dexmedetomidine hydrochloride nasal spray [cumulative ≤ 3μg/kg (28)] in the experimental group, or half the initial dose of chloral hydrate [maximum 1 g; cumulative ≤ 100 mg/kg (29)] in the control group. If sedation remains inadequate, phenobarbital sodium (6 mg/kg, maximum 0.1 g, intravenously or intramuscularly) will be administered by an anesthesiologist as second-line rescue. After rescue, vital signs and MOAA/S score will be monitored every 5 min, and post-procedure observation will be extended to at least 2 h.

#### 2.6.5 Concomitant therapy

To minimize the impact on the routine medical behavior of the children, there are no special restrictions on the children receiving other relevant medical measures during the study period. All concomitant therapies received by the participants will be recorded in detail in the case report form (CRF) for subsequent subgroup analysis.

### 2.7 Outcome measures

#### 2.7.1 Primary outcome measure

Sedation success rate: the proportion of children who achieve the desired depth of sedation and successfully complete relevant diagnostic and therapeutic procedures with a single dose of dexmedetomidine hydrochloride nasal spray (2μg/kg) or oral chloral hydrate (50 mg/kg) without the need for rescue drugs. Children who receive any rescue medication (first-line supplementation or second-line phenobarbital), regardless of whether the procedure is ultimately completed, will not be counted as successful in the primary outcome. The Modified Observer's Assessment of Alertness and Sedation (MOAA/S) scale is used to evaluate the depth of sedation, and a MOAA/S score ≤ 2 indicates that the depth of sedation is sufficient for diagnostic and therapeutic procedures (Table 1). The MOAA/S scale is widely used in pediatric procedural sedation with good inter-rater reliability. A score ≤ 2 corresponds to moderate-to-deep sedation, which ensures the immobility required for all included examinations (MRI, CT, echocardiography, ABR, ophthalmic examinations, etc.) while preserving spontaneous breathing. A uniform threshold was adopted to ensure objective and consistent outcome assessment across procedures (30, 31).

**Table 1:** MOAA/S scale criteria.

Grade	Responsiveness
6	Awake appearance, prompt response to name called in normal tone
5	Appears asleep, prompt response to name called in normal tone
4	Sluggish response to name called in normal tone
3	Response only after name called loudly or repeatedly
2	Response only after mild prodding or shaking
1	No response to mild prodding or shaking
0	No response to any stimulation

#### 2.7.2 Secondary outcome measures

1. Rescue sedation success rate: the proportion of children who achieve the desired depth of sedation and successfully complete the diagnostic or therapeutic procedures after rescue measures, reported separately as a secondary outcome. The initiation criteria, rescue regimen and monitoring requirements are described in Section 2.6.4.
2. Onset time of sedation: the time from initial administration to reaching the desired depth of sedation (MOAA/S score ≤ 2).
3. Duration of sedation: a key indicator reflecting the efficacy of sedative drugs, which is the time from the onset of sedation (MOAA/S score ≤ 2) to the child's recovery (Steward recovery score reaching 4 points). The Steward recovery scale includes three items: activity, respiration and consciousness, with a score of 0-2 for each item (Table 2).
4. Failure rate of diagnostic and therapeutic procedures: the proportion of children who cannot complete the diagnostic and therapeutic procedures due to insufficient sedation (MOAA/S score still > 2) even after rescue measures, serious adverse events or termination of the scheduled diagnostic and therapeutic procedures due to the child's physiological indicators exceeding the threshold.
5. Delayed recovery rate: the proportion of children with a sedation recovery time exceeding 120 min. The time from the last administration of sedative drugs to complete recovery (Steward recovery score reaching 4 points) is defined as sedation recovery time, and a recovery time exceeding 120 min is considered delayed recovery.

#### 2.7.3 Safety evaluation

Vital signs including heart rate (HR), oxygen saturation (SpO_2_), respiratory rate (RR), blood pressure (BP) and MOAA/S score will be monitored and recorded before administration (baseline) and every 5 min after administration to evaluate the sedation effect and safety, which is responsible for assessment, recording and management by anesthesiologists. The Steward recovery score is evaluated every 30 min during the sedation recovery period.

**Table 2:** Steward recovery scale criteria.

Item	Steward Score	Score (Points)
Activity	Purposeful movement of limbs	2
	Non-purposeful movement of limbs	1
	No limb movement	0
Respiration	Coughing on command	2
	Patent airway without respiratory support	1
	Requiring respiratory support	0
Consciousness	Fully awake	2
	Responsive to stimulation	1
	Unresponsive to stimulation	0

#### 2.7.4 Primary adverse events

Adverse events are defined by dual criteria—relative change from baseline or age-specific absolute thresholds (Pediatric Advanced Life Support guidelines) (32), whichever is met: bradycardia (HR decrease ≥ 20% from baseline, or < 80 beats/min for 2–3 years and < 70 beats/min for > 3–6 years); hypotension (SBP decrease ≥ 20% from baseline, or < 70 + age  ×  2 mmHg); hypertension (SBP increase ≥ 20% from baseline); hypoxemia (SpO_2_ < 90% for > 10 s, or decrease ≥ 10% from baseline for > 10 s in cyanotic congenital heart disease); apnea (> 20 s); respiratory intervention (manual ventilation or artificial airway). Baseline values are taken in a calm state, and all events are assessed and managed by anesthesiologists.

#### 2.7.5 Other adverse events

Nausea, vomiting and hypersensitivity reactions, etc.

After the examination, children who received rescue medication will be observed for at least 2 h, and other children for at least 1 h, and the parents will be informed of the discharge precautions after the children's vital signs are stable and their consciousness and behavior return to normal. A 24-hour follow-up call will be conducted for all participants to record any late-onset adverse events after discharge.

#### 2.7.6 Definition, evaluation and management of adverse events

An adverse event (AE) refers to any adverse medical event occurring after drug administration, which is not necessarily causally related to the drug; a serious adverse event (SAE) includes events such as death, life-threatening conditions, disability, need for hospitalization or prolongation of hospitalization time, and congenital malformations. The causal relationship between AE and the drug is evaluated according to a five-level standard from “definitely unrelated” to “definitely related”, among which “definitely related” and “possibly related” are counted as adverse drug reactions. The severity of AE is divided into grades 1–5 according to the CTCAE 5.0 standard. All AEs must be recorded in detail in the case report form (CRF). General AEs should be reported to the sponsor's pharmacovigilance department and the Ethics Committee within 7 working days after being informed, and SAEs must be reported within 24 h. All AEs must be followed up until the event subsides, laboratory values return to normal, return to baseline, loss to follow-up or death. All adverse events will be reported as counts and percentages by group, and sedation success rates will be reported both overall and stratified by examination type.

#### 2.7.7 Termination or adjustment of interventions

If the continued implementation of the study protocol may endanger the safety of the children, or the parents withdraw the informed consent form, or the data reliability is impaired, the participants will stop the intervention and withdraw from the study. Researchers need to respond flexibly to ensure the scientificity and ethicality of the study. The criteria and process for terminating the study for individual participants or the entire study will be reviewed and confirmed by the independent data monitoring committee (IDMC) regularly.

#### 2.7.8 Data collection and recording

Data including age, body weight, gender, ASA physical status classification, examination type (with specific procedure and actual examination duration), main diagnosis, sedation success rate, rescue sedation status, onset time and duration of sedation, actual administered doses of study drugs and adverse events of the children will be collected in this study. All data are entered and managed using the case report form (CRF), which is ensured to be derived from the source documents of the clinical trial to guarantee its accuracy, completeness, readability and timeliness. Modifications to the source data must be traceable and the reasons must be indicated. The principal investigator will assign a special person to be responsible for CRF entry and supervise the data quality. All CRF data will be double-checked by two independent researchers to reduce entry errors, and the electronic data capture (EDC) system will be used for data management with real-time data validation rules.

### 2.8 Statistical analysis

Statistical analyses will be performed under blinded conditions, with group allocation coded as Group A/Group B until the analysis plan is locked. This study will use the intention-to-treat (ITT) principle for result analysis, including all randomly assigned participants. A per-protocol (PP) analysis will be conducted as a sensitivity analysis, excluding participants who violate the study protocol or have major missing data. Measurement data are expressed as mean ± standard deviation, and the independent samples t-test is used for comparison between groups. Measurement data that do not conform to the normal distribution are expressed as median and interquartile range, and the Mann–Whitney U test is used for comparison between groups. Count data are expressed as percentage, and the chi-square test is used for comparison between groups; if the chi-square test is not applicable, the Fisher's exact probability test is used. Statistical analysis is performed using R software, and the statistical significance is set at *p* < 0.05. For repeated measurement data (including HR, SpO_2_, RR, BP and MOAA/S score), a mixed-effects model is used to analyze the differences in changes over time between the two groups. Missing data of primary and secondary outcomes are not imputed, and analysis is directly performed based on the actual observed values.

Subgroup analyses will be performed according to examination type (CT, MRI, and others) to compare sedation success rates between the two groups across procedures with different durations; logistic regression will be used to adjust for examination duration, age, and body weight as covariates. In addition, subgroup analyses by age (2–3 years vs. 4–6 years) and body weight will be conducted to examine the robustness of treatment effects across these subgroups (33).

Safety equivalence will be concluded if the 95% confidence interval of the between-group difference in adverse event incidence lies entirely within −15% to +15%.

No interim analysis will be conducted in this study.

### 2.9 Patient and public involvement

This study protocol has been approved by the Medical Ethics Committee of the Affiliated Hospital of Southwest Medical University (Approval No.: KY2025368, Approval Date: Sep. 25th, 2025). All procedures involving human participants are in accordance with the Declaration of Helsinki (2013 revision). Written informed consent will be obtained from the legal guardians of all participating children before enrollment, and the guardians have the right to withdraw from the study at any time without any impact on the clinical treatment of the children. All researchers and participants have been notified of the protocol revisions, and participants have subsequently signed the updated informed consent forms. The corresponding amendments have also been updated on the clinical trial registration platform. All clinical statistical data will be securely stored by two designated researchers. Our findings will be published in a peer-reviewed scientific journal.

## 3 Discussion

This study is a single-center, prospective, randomized controlled trial designed to compare the clinical efficacy and safety of dexmedetomidine hydrochloride nasal spray and oral chloral hydrate for pre-examination sedation in children aged 2 to 6 years. From the perspective of study design, the randomized controlled method is adopted to ensure the comparability between the two groups, and conducting the study under routine clinical conditions makes the results more applicable to daily clinical practice. The sample size is determined by comprehensive consideration of efficacy and safety endpoints, and the sample size calculation is scientific and reasonable, which can ensure the statistical power of the study.

In terms of intervention measures, dexmedetomidine hydrochloride nasal spray is selected as the experimental drug, which makes up for the shortcomings of traditional sedation methods such as oral chloral hydrate. Its intranasal administration route has the advantages of rapid absorption, no first-pass effect, convenient operation and good tolerance, which is more suitable for young children who are difficult to cooperate with oral or injection administration. The dose of 2μg/kg is determined based on existing literature reports and clinical experience, which is a safe and effective dose range for pediatric intranasal sedation.

In terms of outcome measures, the sedation success rate is set as the primary outcome measure, and the MOAA/S scale with high clinical applicability is used to evaluate the depth of sedation, which can accurately reflect the sedation effect of the drug. Secondary outcome measures include rescue sedation success rate, onset time and duration of sedation, failure rate of diagnostic and therapeutic procedures and delayed recovery rate, which comprehensively evaluate the efficacy of the drug from multiple dimensions. The safety evaluation includes comprehensive monitoring of vital signs and close observation of adverse events, and the definition and grading standards of adverse events are clear, which can accurately evaluate the safety of the drug.

There are also some limitations in this study. First, this is a single-center study, and the study results may be affected by the clinical characteristics of the single center, so the external validity of the results needs to be verified by multi-center studies. Second, the study only includes children aged 2 to 6 years with ASA physical status classification I or II, and the results are not applicable to children with more severe systemic diseases or younger/older age groups. Third, the follow-up time of the study is short, and the long-term effects of dexmedetomidine hydrochloride nasal spray on the neurological development of young children cannot be evaluated, which needs to be further studied by long-term follow-up studies. Fourth, blinding of the children and the sedation nurses was not feasible due to the distinct administration routes, which may introduce performance bias. However, assessor blinding was implemented to minimize detection and assessment bias.

This study is designed to generate randomized evidence for a widely practiced clinical model: procedural sedation—rather than general anesthesia—is the standard approach for outpatient imaging in ASA I/II children in most Chinese pediatric centers, yet high-quality randomized evidence comparing intranasal dexmedetomidine with oral chloral hydrate in this setting remains limited. It should also be noted that the sedative efficacy of chloral hydrate may vary with age (33); the prespecified age and body weight subgroup analyses will help clarify this issue.

In conclusion, this study will systematically compare the clinical efficacy and safety of dexmedetomidine hydrochloride nasal spray with oral chloral hydrate for pre-examination sedation in children aged 2 to 6 years. Regardless of the direction of the results, the findings will provide high-quality evidence for sedation strategies in this population, enrich the clinical application of intranasal sedation drugs in pediatrics, and have important clinical significance for improving the diagnostic experience of young children and reducing medical psychological trauma.
